# Supplementary material for: Novel Driver Strength Index highlights important cancer genes in TCGA PanCanAtlas patients
Source: PeerJ. 2022 Aug 11;10:e13860. doi: 10.7717/peerj.13860 (PMC9375969; doi:10.7717/peerj.13860)
Supplement: Supplemental Information 2 [file peerj-10-13860-s002.zip › COHORTS/genes plots/cohorts/2021_8_16_14_9_distribution_cohorts_males_MESO.pdf]

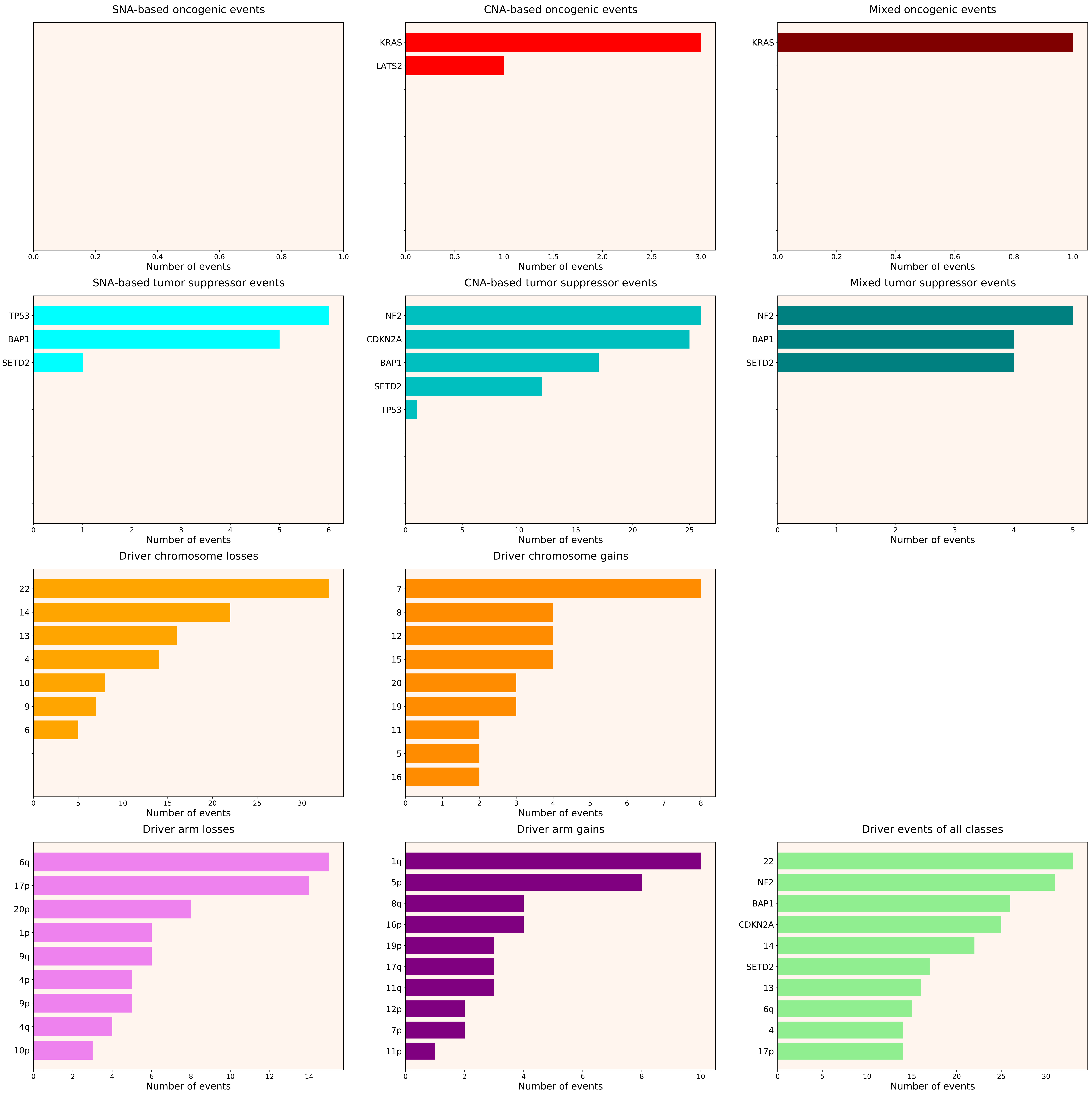
SNA-based oncogenic events
0.0
0.2
0.4
0.6
0.8
1.0
Number of events
CNA-based oncogenic events
KRAS
LATS2
0.0
0.5
1.0
1.5
2.0
2.5
3.0
Number of events
Mixed oncogenic events
KRAS
0.0
0.2
0.4
0.6
0.8
1.0
Number of events
SNA-based tumor suppressor events
TP53
BAP1
SETD2
0
1
2
3
4
5
6
Number of events
CNA-based tumor suppressor events
NF2
CDKN2A
BAP1
SETD2
TP53
0
5
10
15
20
25
Number of events
Mixed tumor suppressor events
NF2
BAP1
SETD2
0
1
2
3
4
5
Number of events
Driver chromosome losses
22
14
13
4
10
9
6
0
5
10
15
20
25
30
Number of events
Driver chromosome gains
7
8
12
15
20
19
11
5
16
0
1
2
3
4
5
6
7
8
Number of events
Driver arm losses
6q
17p
20p
1p
9q
4p
9p
4q
10p
0
2
4
6
8
10
12
14
Number of events
Driver arm gains
1q
5p
8q
16p
19p
17q
11q
12p
7p
11p
0
2
4
6
8
10
Number of events
Driver events of all classes
22
NF2
BAP1
CDKN2A
14
SETD2
13
6q
4
17p
0
5
10
15
20
25
30
Number of events
